# Presence of Lassa Virus RNA in Cerebrospinal Fluid Indicating Neuroinvasive Lassa Fever in Pediatric Patients From Edo State, Nigeria

**DOI:** 10.1093/infdis/jiaf547

**Published:** 2025-11-25

**Authors:** Hannah Caroline Sophie Müller, Cyril Oshomah Erameh, Joseph Okoeguale, Sheila Ojor Ileli, Imonifome Frank Onyeke, Adewale Elijah Adetunji, Lilian Omoyemen Akerele, Rita Esumeh, Ebo Benevolence Ohomoime, Mette Hinrichs, Jonas Müller, Ujiagbe Moses Aiterebhe, Christiana Ngozi Ekuma, Chukwuemeka Ogbuinya Ugadu, Ifeanyi Henry Onyerikam, Juliet Oemhenze Idialu-Eigbobo, Matthew Apeleokha, Ehisuan Ehiaghe, Osahogie Isaac Edeawe, Kelly Ohis Iraoyah, Chris Hoffmann, Donatus Adomeh, Thomas Olokor, Ikponmwosa Odia, Danny Asogun, Sylvanus Okogbenin, Ephraim Ogbaini-Emovon, Reuben Eifediyi, Stephan Günther, Meike Pahlmann, Michael Ramharter, Lisa Oestereich, Till Omansen, George Akpede

**Affiliations:** Department of Tropical Medicine, Bernhard Nocht Institute for Tropical Medicine and I. Department of Medicine, University Medical Center Hamburg-Eppendorf, Hamburg, Germany; Department of Virology, Bernhard Nocht Institute for Tropical Medicine, Hamburg, Germany; Institute of Viral and Emergent Pathogens Control and Research, Irrua Specialist Teaching Hospital, Irrua, Nigeria; Department of Medicine, Irrua Specialist Teaching Hospital, Irrua, Nigeria; Institute of Viral and Emergent Pathogens Control and Research, Irrua Specialist Teaching Hospital, Irrua, Nigeria; Department of Obstetrics and Gynecology, Irrua Specialist Teaching Hospital, Irrua, Nigeria; Department of Pediatrics, Irrua Specialist Teaching Hospital, Irrua, Nigeria; Department of Pediatrics, Irrua Specialist Teaching Hospital, Irrua, Nigeria; Department of Pediatrics, Irrua Specialist Teaching Hospital, Irrua, Nigeria; Department of Pediatrics, Irrua Specialist Teaching Hospital, Irrua, Nigeria; Institute of Viral and Emergent Pathogens Control and Research, Irrua Specialist Teaching Hospital, Irrua, Nigeria; Institute of Viral and Emergent Pathogens Control and Research, Irrua Specialist Teaching Hospital, Irrua, Nigeria; Department of Virology, Bernhard Nocht Institute for Tropical Medicine, Hamburg, Germany; Department of Virology, Bernhard Nocht Institute for Tropical Medicine, Hamburg, Germany; Institute of Viral and Emergent Pathogens Control and Research, Irrua Specialist Teaching Hospital, Irrua, Nigeria; Department of Pediatrics, Irrua Specialist Teaching Hospital, Irrua, Nigeria; Department of Pediatrics, Irrua Specialist Teaching Hospital, Irrua, Nigeria; Department of Pediatrics, Irrua Specialist Teaching Hospital, Irrua, Nigeria; Department of Pediatrics, Irrua Specialist Teaching Hospital, Irrua, Nigeria; Department of Pediatrics, Irrua Specialist Teaching Hospital, Irrua, Nigeria; Department of Pediatrics, Irrua Specialist Teaching Hospital, Irrua, Nigeria; Institute of Viral and Emergent Pathogens Control and Research, Irrua Specialist Teaching Hospital, Irrua, Nigeria; Department of Medicine, Irrua Specialist Teaching Hospital, Irrua, Nigeria; Department of Virology, Bernhard Nocht Institute for Tropical Medicine, Hamburg, Germany; Institute of Viral and Emergent Pathogens Control and Research, Irrua Specialist Teaching Hospital, Irrua, Nigeria; Institute of Viral and Emergent Pathogens Control and Research, Irrua Specialist Teaching Hospital, Irrua, Nigeria; Institute of Viral and Emergent Pathogens Control and Research, Irrua Specialist Teaching Hospital, Irrua, Nigeria; Department of Community Medicine, Irrua Specialist Teaching Hospital, Irrua, Nigeria; Institute of Viral and Emergent Pathogens Control and Research, Irrua Specialist Teaching Hospital, Irrua, Nigeria; Department of Obstetrics and Gynecology, Irrua Specialist Teaching Hospital, Irrua, Nigeria; Department of Microbiology, Irrua Specialist Teaching Hospital, Irrua, Nigeria; Department of Obstetrics and Gynecology, Irrua Specialist Teaching Hospital, Irrua, Nigeria; Department of Virology, Bernhard Nocht Institute for Tropical Medicine, Hamburg, Germany; German Center for Infection Research, partner sites Hamburg, Lübeck, Borstel, Riems, Germany; Department of Virology, Bernhard Nocht Institute for Tropical Medicine, Hamburg, Germany; Department of Tropical Medicine, Bernhard Nocht Institute for Tropical Medicine and I. Department of Medicine, University Medical Center Hamburg-Eppendorf, Hamburg, Germany; German Center for Infection Research, partner sites Hamburg, Lübeck, Borstel, Riems, Germany; Department of Virology, Bernhard Nocht Institute for Tropical Medicine, Hamburg, Germany; German Center for Infection Research, partner sites Hamburg, Lübeck, Borstel, Riems, Germany; Department of Tropical Medicine, Bernhard Nocht Institute for Tropical Medicine and I. Department of Medicine, University Medical Center Hamburg-Eppendorf, Hamburg, Germany; Department of Virology, Bernhard Nocht Institute for Tropical Medicine, Hamburg, Germany; German Center for Infection Research, partner sites Hamburg, Lübeck, Borstel, Riems, Germany; Institute of Viral and Emergent Pathogens Control and Research, Irrua Specialist Teaching Hospital, Irrua, Nigeria; Department of Pediatrics, Irrua Specialist Teaching Hospital, Irrua, Nigeria; Department of Pediatrics, Faculty of Clinical Sciences, College of Medicine, Ambrose Alli University, Ekpoma, Nigeria

**Keywords:** Lassa fever, Lassa virus, LASV, CSF, meningoencephalitis

## Abstract

**Background:**

Neurological complications of Lassa fever (LF) are associated with fatal outcome. In this study, we aimed to provide further evidence of Lassa virus (LASV) infection of the central nervous system (CNS) by assessing LASV in cerebrospinal fluid (CSF).

**Methods:**

We retrospectively screened the database of the LF diagnostic unit at Irrua Specialist Teaching Hospital in Nigeria for patients with suspected or confirmed LF who underwent lumbar puncture as part of their routine clinical management due to CNS symptoms and had CSF samples tested by LASV reverse-transcription polymerase chain reaction (RT-PCR).

**Results:**

RT-PCR results for CSF were available for 153 patients, all children. LF was confirmed in 49 of 153 (32%) patients, of whom 42 (86%) were LASV RNA positive in CSF. Of the 42 patients, 33 (79%) were LASV RNA positive in CSF and plasma, whereas 9 (21%) patients were positive in CSF only. The CSF-positive LF patients had a median age of 10.5 years. Sample pairs of CSF and plasma taken within a day of each other on admission were available for 26 patients, of whom 23 (88%) had higher LASV RNA concentration in CSF compared to plasma (cycle threshold, 28.2 vs 36.7, respectively; *P* < .00001).

**Conclusions:**

LASV is frequently detected in CSF of pediatric LF patients with neurological symptoms. The virus load in CSF is usually higher than in plasma, indicating a neuroinvasive infection with active virus replication in CNS. Our findings have implications for clinical management of LF patients and drug development for LF.

## Teaser

The cerebrospinal fluid (CSF) of 86% of pediatric Lassa fever patients who underwent lumbar puncture due to central nervous system symptoms was Lassa virus (LASV) RT-PCR positive. When comparing plasma and CSF samples, the concentration of LASV RNA was significantly higher in CSF than in plasma.

**(See the Editorial Commentary by Platt and Chertow on pages e593–5.)**

Lassa fever (LF), caused by Lassa virus (LASV), is a viral hemorrhagic fever endemic in West Africa, particularly in Nigeria, Sierra Leone, and Guinea. Approximately 100 000–300 000 infections are estimated to occur every year [1]. LF is listed as a priority disease in the World Health Organization Research and Development Blueprint for Epidemics because no licensed vaccine is available; hospitalized cases show high case fatality of 15%–30% [2, 3]; and treatment options are limited to off-label use of ribavirin, despite varying evidence for its efficacy [4, 5], and supportive care.

Severe LF may cause neurological complications, such as meningitis, seizures, hearing loss, and other focal deficits as well as altered mental state [6–13], which are strongly associated with fatal disease outcome [10, 14–16]. Despite this, it remains unknown whether LASV directly infects the central nervous system (CNS) and how neurological complications arise. While seizures, confusion, restlessness, and altered mental state may result from metabolic encephalopathy, meningitis and focal deficits suggest direct CNS involvement.

Thus far, LASV RNA [13, 17, 18] or infectious virus [16] has reportedly been detected in CSF of only a few isolated cases presenting with symptoms of (meningo-) encephalitis, such as fever, clinical meningitis, lethargy, hearing loss, dysarthria and dysphagia [18], persistent fever and headache [13], fever, disorientation, somnolence, and seizure [17]. Notably, in 2 patients, LASV RNA was found in cerebrospinal fluid (CSF) but not in blood [13, 17]. Therefore, replication of the virus within the CNS has been discussed as a pathophysiological mechanism for neurological findings similar to Ebola virus disease [19]. It remains unclear whether these findings are incidental or whether LASV may be detected more consistently in CSF.

Other members of the Arenaviridae family, such as Junín virus (JUNV) and lymphocytic choriomeningitis virus (LCMV), are known to be neuroinvasive in humans [20, 21]. Dependent on factors such as viral load and strain, route of transmission, and host immunocompetence, different courses of CNS disease from asymptomatic to lifelong persistent infection and fatal outcome have been described for LCMV in animal models [22]. In mice, LCMV reduces the blood-brain barrier (BBB) integrity through cytotoxic CD8^+^ cell activation [22, 23] to enter the CNS and causes ventricular damage with CSF perfusion into the brain parenchyma, increased intracranial pressure, and seizures [22]. For JUNV, a mouse model showed virus transport from the skin entry portal via peripheral nerves to the CNS [24]. Apart from this, high viremia with BBB bypassing was discussed as another possible entry route explaining variable development of neurological symptoms and outcome [24, 25].

To treat LF more efficiently, it is crucial to understand whether LASV infection affects the CNS and, if so, by which pathomechanisms. Indirect effects such as cytokine-induced neuronal dysfunction, metabolic disturbances, microbleeding or microclotting, and endothelial dysfunction are conceivable. However, 2 cases reported so far with LASV RNA detected in CSF but not in blood suggest that active LASV replication in CNS might play a role [13, 17]. In this study, we aimed to provide further evidence that LASV may directly affect the CNS. To this end, we took advantage of the recent introduction of real-time polymerase chain reaction (PCR) in the LF diagnostic laboratory at Irrua Specialist Teaching Hospital (ISTH). The cycle threshold (Ct) readout of real-time PCR as a measure of virus RNA concentration facilitates a quantitative comparison of virus load in CSF versus plasma. We retrospectively screened the database of the LF diagnostic unit for patients with suspected or laboratory-confirmed LF who underwent lumbar puncture as part of their routine clinical management and had at least 1 CSF sample tested by LASV reverse-transcription polymerase chain reaction (RT-PCR). We then compared LASV RT-PCR results of CSF and plasma.

## MATERIALS AND METHODS

### Study Design and Data Collection

We conducted a retrospective laboratory-based study at the LF diagnostic laboratory of the Institute of Viral and Emergent Pathogens Control and Research at ISTH, Edo State, Nigeria. ISTH is a national Center of Excellence for LF management and research. It is among the centers with the highest caseload of LF within West Africa. Patients with suspected or laboratory-confirmed LF who underwent lumbar puncture as part of their routine clinical management and had at least 1 CSF sample tested by LASV RT-PCR between 1 January 2021 and 31 October 2023 were included in the study. The decision to send a blood or CSF sample to the laboratory for testing rested solely with the treating physician. A total of 153 pediatric patients meeting these criteria were included in the study. Children with meningism routinely undergo lumbar puncture to test for bacterial meningitis. Table 1 provides a list of major indications for lumbar puncture in children at ISTH, given there are no contraindications [26–29]. A more cautious diagnostic approach is employed at ISTH for adults with meningism and suspected LF due to concerns for clinically relevant bleeding in this population as well as the increased risk of other diseases such as neoplasia.

**Table 1.** Indications for Lumbar Puncture in Pediatric Patients With Suspected or Confirmed Lassa Fever at Irrua Specialist Teaching Hospital^a^

Indications for Lumbar Puncture
Presence of typical signs of meningeal irritation: nuchal rigidity, positive Kerning and Budzinski signs at any age; bulging, tense fontanelle in infants [28]
Headache and/or photophobia associated with fever in children >3 years of age irrespective of the presence of meningeal signs
Irritability and excessive and unconsolable crying in infants and toddlers with fever irrespective of the presence of meningeal signs
Febrile status epilepticus
Exclusion of meningitis in children <2 y of age with simple or complex febrile convulsions [26, 27]
Exclusion of meningitis in children aged ≥2 y with complex febrile seizures
Exclusion of meningitis in children with alteration of consciousness and fever with or without convulsions [29]
Exclusion of LF through testing of CSF for LASV in plasma LASV-negative children in whom there is a high suspicion for LF
Monitoring of response to LF treatment/appearance of new clinical features during treatment

Abbreviations: CSF, cerebrospinal fluid; LASV, Lassa virus; LF, Lassa fever.

^a^The table describes indications of lumbar puncture in the context of LASV suspicion at our study site, Irrua Specialist Teaching Hospital in Edo State, Nigeria, to show in which constellations children with suspected LF received CSF analysis and were subsequently included in our study.

In the LF diagnostic laboratory, viral RNA was extracted from CSF and plasma samples using the QIAamp Viral RNA Mini Kit (Qiagen). RT-PCR was conducted using the RealStar Lassa Virus RT-PCR Kit 2.0 (Altona Diagnostics). The RT-PCR assay comprises 2 different reactions targeting the viral S and L genome segments, respectively. Ct values, as a proxy of virus RNA concentration, were recorded for both targets.

The patients’ symptoms and demographical data were reported by clinicians on paper-based laboratory request forms. Together with RT-PCR results, this information was recorded in an electronic database of the laboratory. From here, relevant study information was extracted, de-identified, and compiled into a single spreadsheet for further analysis.

### Statistical Analysis

Using descriptive statistics, we analyzed demographic and clinical characteristics of CSF-positive and -negative LF patients. To compare the Ct values for CSF and plasma in the same patient, CSF/plasma sample pairs were defined, collected within 1 day of each other. To exclude potential treatment effects of ribavirin, only paired samples taken on admission were included. Wilcoxon signed-rank test was used to test for significant differences between paired samples, Mann–Whitney U test for unpaired samples, and χ^2^ test or Fisher exact test for proportions. A *P* value of .05 with Bonferroni correction for multiple testing was considered significant. For nonparametric statistical testing, negative RT-PCR results were given a Ct of 45, above the measurement range of the assay. For 2 patients with multiple follow-up samples of plasma and CSF tested by RT-PCR, the Ct kinetics were analyzed longitudinally. The statistical analysis was performed using RStudio 2023.12.1+402, and plots were created with GraphPad Prism 10.2.3.

### Ethics Statement

As this study followed a retrospective design with no harm to patients or their privacy due to de-identification of personal data, an exemption from ethical approval and requirement of patient consent was granted by the local ethics committee at ISTH.

## RESULTS

### Study Population

**Figure 1.**
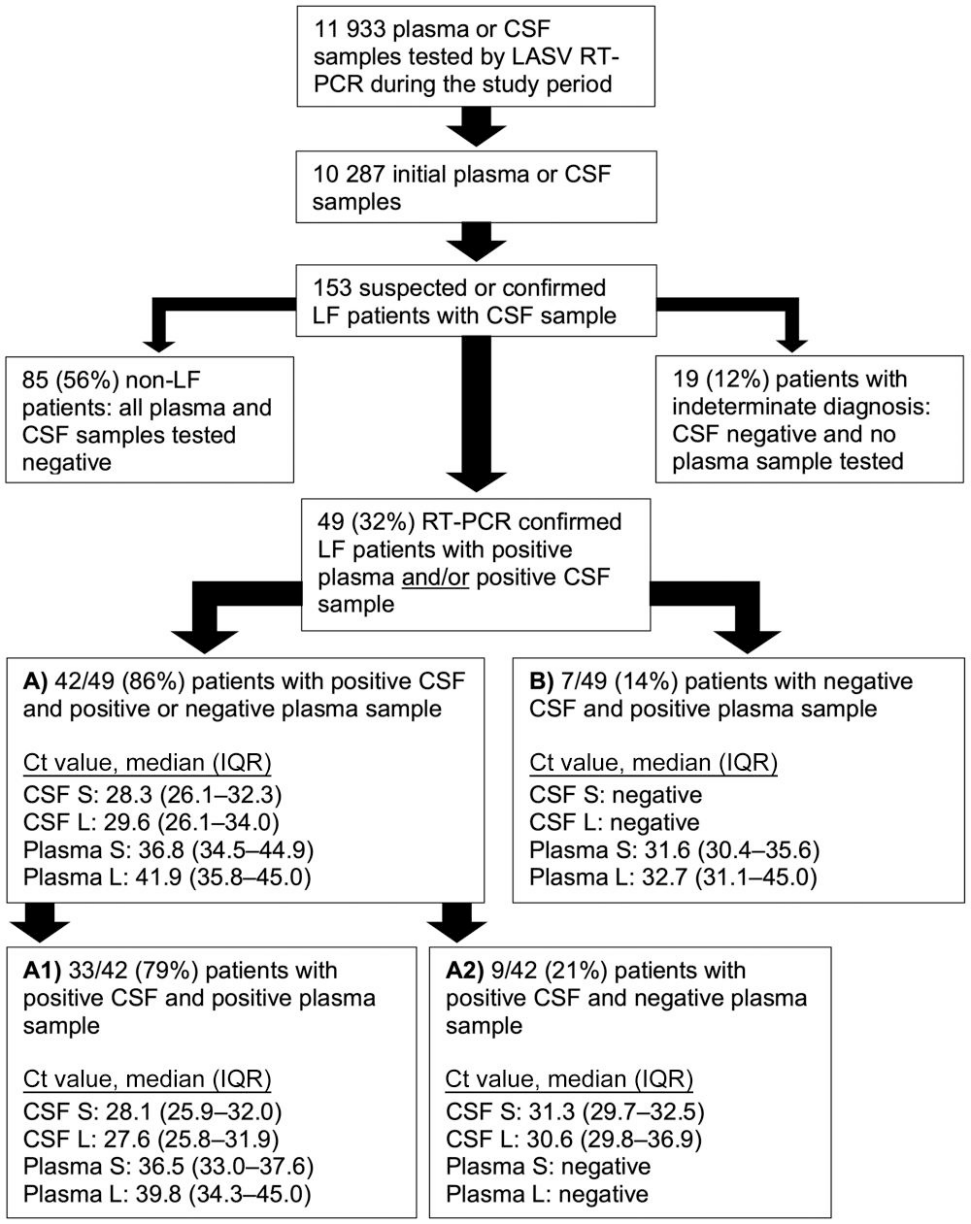
Flowchart of data analysis. The study population comprised all patients with suspected or confirmed Lassa fever with at least 1 cerebrospinal fluid (CSF) sample tested by Lassa virus reverse-transcription polymerase chain reaction (RT-PCR). The RT-PCR results for the different subgroups are indicated. Whenever multiple CSF or plasma samples were available per patient due to follow-up testing, only the initial samples are illustrated to ensure better comparability. Abbreviations: CSF, cerebrospinal fluid; Ct, cycle threshold; IQR, interquartile range; L, reverse-transcription polymerase chain reaction Lassa virus L segment target; LASV, Lassa virus; LF, Lassa fever; RT-PCR, reverse-transcription polymerase chain reaction; S, reverse-transcription polymerase chain reaction Lassa virus S segment target.

During the study period, 11 933 plasma or CSF samples were tested by LASV RT-PCR at ISTH, of which 10 287 were initial samples, that is, the first plasma sample taken to confirm the diagnosis and the first CSF sample taken during hospitalization. Among the initial samples, 153 were CSF samples originating from 153 patients (Figure 1). The diagnosis of LF was confirmed by RT-PCR in 49 of 153 (32%) patients with CSF tested. The 49 patients with LF could further be subdivided into 2 groups according to the CSF findings: (A) 42 of 49 (86%) patients tested LASV RNA positive in CSF, and (B) 7 of 49 (14%) patients tested negative in CSF but had positive results for plasma. Most patients in group A (33/42 [79%]) tested LASV RNA positive in both CSF and plasma, while 9 of 42 (21%) patients were CSF positive but their plasma samples were negative. Thus, the majority of LF patients who underwent lumbar puncture due to CNS symptoms had LASV RNA in CSF. In about 20% of these patients, plasma samples tested negative, that is, the diagnosis of LF was confirmed by CSF testing only.

LF was not confirmed in 85 of 153 (56%) patients with CSF samples tested; they tested negative in all CSF and plasma samples. In 19 of 153 (12%) patients, LF could not be ruled out with certainty; they were negative in all CSF samples, while PCR for plasma was not conducted (Figure 1).

### Demographic and Clinical Characteristics

To explore whether LASV RNA detection in CSF might be associated with demographic or clinical features, the 42 LF patients with LASV RNA–positive CSF were compared with the 7 LF patients with negative CSF as well as with non-LF patients who tested negative in both CSF and plasma (Table 2). The median age of the CSF-positive LF cases was 10.5 years, while the CSF-negative, plasma-positive LF patients were slightly older (median age, 13.0 years; *P* = .38). Forty-three percent of both groups were female. Non-LF patients had similar sex distribution but were considerably younger than CSF-positive LF patients (*P* = .04). There were no significant differences in the timespan from symptom onset to first CSF RT-PCR test between the 3 groups. The fatality rate was 7% for the CSF-positive cases, whereas none of the CSF-negative LF patients had fatal outcome. No significant differences were seen with respect to case fatality among the 3 groups.

**Table 2.** Comparison of Demographic and Clinical Characteristics of Patients Who Underwent Lumbar Puncture for Lassa Virus Reverse-Transcription Polymerase Chain Reaction Testing^a^

Characteristic	Group A: CSF-Positive LF Patients (n = 42)	Group B: CSF-Negative LF Patients (n = 7)	*P* Value (A vs B)	Group C: CSF- and Plasma-Negative Non-LF Patients (n = 85)	*P* Value (A vs C)
Demographic data					
Sex					
Female	18 (43)	3 (43)	1	37 (44)	.91
Male	24 (57)	4 (57)		48 (56)	
Age, y, median (IQR)	10.5 (6.3–14)	13.0 (11.8–14.8)	.38	5.0 (1.8–12.3)	.04
Time from symptom onset to CSF PCR, d, median (IQR)	11.0 (7.0–16.5)	14.5 (10.8–23.5)	.11	4.0 (2.0–9.0)	1
Clinical characteristics					
Symptoms on admission					
Fever	41 (98)	7 (100)	1	70 (82)	.01
Headache	32 (76)	4 (57)	.41	38 (45)	.006
Abdominal pain	32 (76)	5 (71)	1	25 (29)	<.0001
Vomiting	27 (64)	4 (57)	1	28 (33)	.006
General weakness	20 (48)	3 (43)	1	27 (32)	.16
Cough	11 (26)	1 (14)	.66	15 (18)	.24
Chest or retrosternal pain	6 (14)	2 (29)	.58	8 (9)	.38
Sore throat	5 (12)	1 (14)	.55	3 (4)	.35
Bleeding, any type	4 (10)	1 (14)	1	1 (1)	.02
Diarrhea	3 (7)	2 (29)	.20	12 (14)	.55
Seizure	1 (2)	2 (29)	.07	2 (2)	1
Dyspnea	2 (4)	0 (0)	1	10 (12)	.51
Outcome					
Death	3 (7)	0 (0)	1	4 (5)	.40

Data are presented as No. (%) unless otherwise indicated.

Abbreviations: CSF, cerebrospinal fluid; IQR, interquartile range; LF, Lassa fever; PCR, polymerase chain reaction.

^a^The central nervous system symptoms or indications (Table 1) that led to lumbar puncture in a specific patient are not included here, as they were not recorded in the laboratory database. Patients with an indeterminate diagnosis due to missing plasma reverse-transcription PCR data were excluded. Statistical significance of differences between LF patients with Lassa virus (LASV) RNA in CSF vs those without LASV RNA in CSF and vs non-LF patients was tested for by χ^2^ test, Fisher exact test, or Mann–Whitney *U* test. A Bonferroni-corrected *P* value of .003 was considered significant.

The most frequently reported symptoms of CSF-positive patients on admission were fever, headache, abdominal pain, vomiting, and general weakness (Table 2). The symptoms of CSF-negative LF patients differed in frequency, but none of the differences were statistically significant due to their small sample size. Non-LF patients generally reported fewer symptoms than CSF-positive LF patients, with only abdominal pain being statistically significant (*P* < .0001). The clinicians did not report photophobia, hearing loss, disorientation, or coma on the laboratory request forms for any patient. However, in addition to the symptoms in Table 2, the pediatric patients had 1 or several of the CNS symptoms listed as indications for lumbar puncture in Table 1. Which of the indication(s) led to the CSF testing was not recorded in the laboratory database.

### Comparative Analysis of Viral Load in CSF and Plasma

The 42 CSF-positive LF patients were first analyzed regardless of a matching CSF and plasma sample pair. Among all patients, the first plasma RT-PCR was performed a median of 8.5 days after symptom onset and the first CSF RT-PCR 11 days after symptoms commenced. Of the 42 patients, 34 (81.0%) had a lower Ct value in their initial CSF RT-PCR compared to their first plasma sample. Accordingly, median Ct values for the initial CSF RT-PCR of all 42 CSF-positive patients were lower than those in the plasma of this group (Table 3). These data point to a higher viral load in CSF. However, due to the time lapse between sampling time points for plasma and CSF in some patients, no firm conclusions could be drawn.

**Table 3.** Comparison of the Lassa Virus Reverse-Transcription Polymerase Chain Reaction Results for Cerebrospinal Fluid and Plasma

Characteristic	CSF	Plasma	CSF – Plasma Difference
All CSF-positive patients (n = 42)^a^			
Time from symptom onset to date of sampling, d	11.0 (7.0–16.5)	8.5 (4.3–14.0)	1.0 (0–2.0)
Ct value S-target	28.3 (26.1–32.3)	36.8 (34.5–44.9)	−8.2 (−20.8 to −4.5)
Ct value L-target	29.6 (26.1–34.0)	41.9 (35.8–45.0)	−12.5 (−20.3 to −6.1)
Patients with matched CSF and plasma pairs (n = 26)^b^			
Time from symptom onset to date of sampling, d	10.5 (8.8–15.0)	10.0 (8.0–14.0)	0 (0–1.0)
Ct value S-target^c^	28.2 (25.4–30.1)	36.7 (34.5–43.8)	−8.1 (−12.3 to −6.3)
Ct value L-target^d^	29.3 (25.6–30.5)	43.7 (35.8–45.0)	−12.5 (−14.6 to −7.3)

Data are presented as median (interquartile range).

Abbreviations: CSF, cerebrospinal fluid; Ct, cycle threshold.

^a^Comparison of the earliest available plasma and CSF reverse-transcription polymerase chain reaction results regardless of the sampling timepoints.

^b^Comparison of CSF and plasma samples collected not more than 1 day apart on admission. ^c^*P* < .00001 for difference between CSF and plasma.

^d^ *P* < .0001 for difference between CSF and plasma. Statistical significance was tested for by Wilcoxon signed-rank test. A Bonferroni-corrected *P* value of .025 was considered significant.

**Figure 2.**
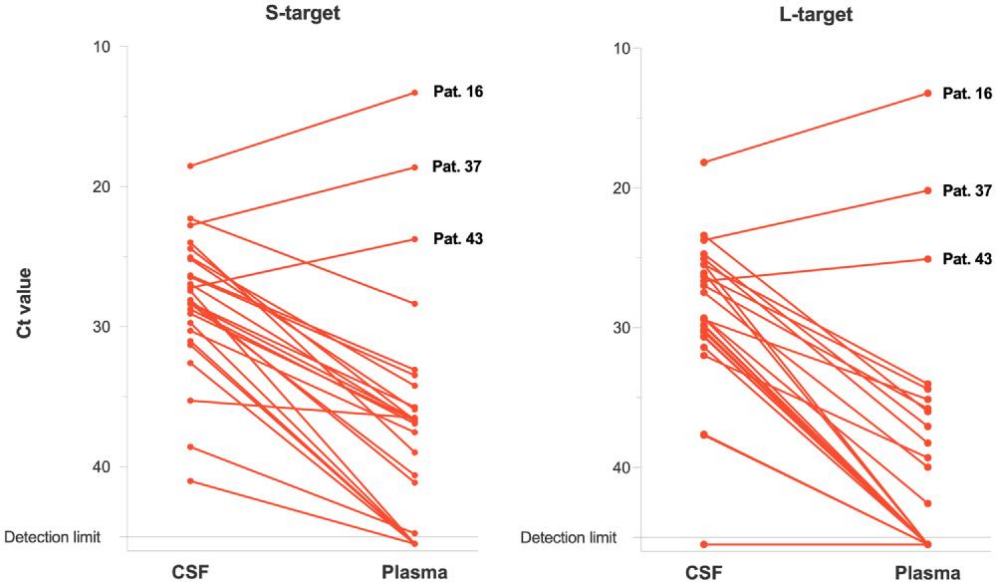
Comparison of Lassa virus cycle threshold (Ct) values between matched pairs of cerebrospinal fluid (CSF) and blood plasma for LASV S- and L-targets. Matched CSF and plasma samples were collected not more than 1 day apart from each other on or shortly after admission. Sample pairs meeting these criteria were identified for 26 patients. Each line represents 1 patient. Negative reverse-transcription polymerase chain reaction results are shown below the detection limit. Three patients with lower Ct value in plasma vs CSF are indicated.

To better compare the viral load between CSF and plasma, we matched CSF and plasma samples collected not more than 1 day apart (Table 3, Figure 2). To exclude potential treatment effects of ribavirin, only paired samples taken on admission were included. For 26 of the 42 patients, sample pairs meeting these criteria were identified. Of the 26 patients, 23 (88%) had a lower Ct value in CSF compared to plasma. Accordingly, the pairs showed significantly lower median Ct values in CSF than in plasma with *P* < .00001 for S-targets and *P* < .0001 for L-targets (Table 3), confirming the results for all 42 patients. The Ct values for S- and L-targets were 8.1 and 12.5 units (median), respectively, lower in CSF compared to plasma (Table 3), which roughly corresponds to a 1000-fold higher LASV RNA concentration in CSF versus plasma. A graphical representation of the differences between CSF and plasma Ct for the 26 paired samples is shown in Figure 2. Apart from 3 patients (numbers 16, 37, and 43) with considerably lower overall Ct values (ie, higher viral load), all other patients had lower Ct values in CSF than in plasma and, hence, a higher concentration of LASV RNA in CSF relative to plasma.

Patients 16, 37, and 43 with higher viral load in plasma than in CSF had a fatal outcome. Patients 16 and 43 were infants with severe LF disease. LF presumably occurred due to intrauterine infection in case of patient 16, whose symptom onset was not precisely recorded, and certainly in case of patient 43, whose symptoms commenced the day after delivery. Both infants underwent same day CSF and plasma RT-PCR tests at the age of 17 and 11 days, respectively. Patient 16 exhibited the lowest Ct values of all patients in CSF (S-target, 18.5; L-target, 18.1) and plasma (S-target, 13.3; L-target, 13.2), while patient 43 also showed very high viral load (CSF: S-target, 22.7 and L-target, 23.7; plasma: S-target, 18.6 and L-target, 20.1). The third fatal case (patient 37) was a 5-year-old girl who presented to ISTH with a 9-day history of fever, abdominal pain, diarrhea, nausea, and vomiting. She died on day of admission before further diagnostic modalities could be initiated.

**Figure 3.**
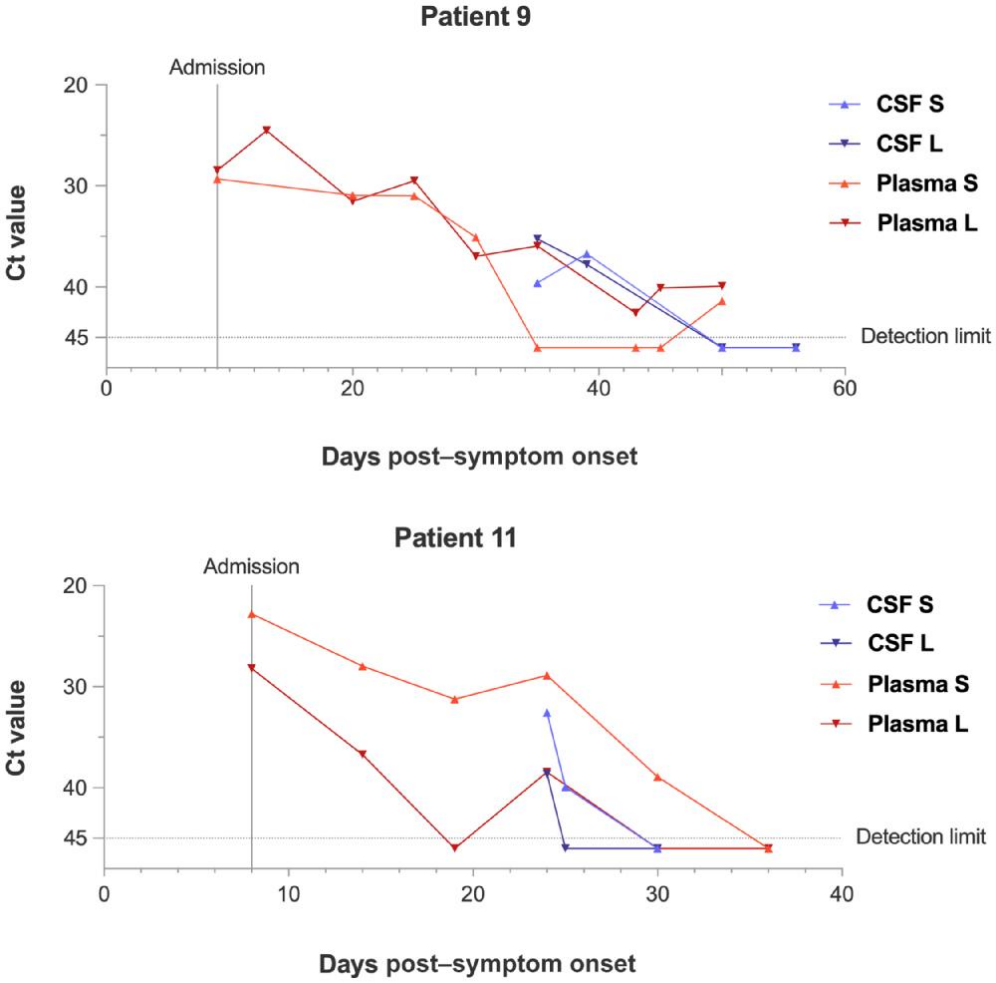
Lassa virus cycle threshold values for cerebrospinal fluid (CSF) and plasma of patients 9 and 11 over the course of disease. Both cases show prolonged detection of viral RNA in plasma with late-stage detection of viral RNA in CSF. Negative reverse-transcription polymerase chain reaction results are shown below the detection limit. Abbreviations: CSF, cerebrospinal fluid; Ct, cycle threshold; L, reverse-transcription polymerase chain reaction Lassa virus L segment target; S, reverse-transcription polymerase chain reaction Lassa virus S segment target.

Multiple RT-PCR results for plasma and CSF were available for 2 patients, showing prolonged LASV RNA detection in plasma over 30 and 50 days after symptom onset (26 and 41 days after admission), respectively, combined with late-stage virus RNA detection in CSF on days 23 and 39, respectively (Figure 3). This duration of virus RNA detection in plasma is substantially longer than in most LF patients treated at ISTH [30]. Because the lumbar punctures were only performed in later disease stages, it remains unclear when the CSF samples initially became positive. Patient 9, a 15-year-old girl, was admitted to ISTH on day 8 post–symptom onset with fever, abdominal pain, and headache. When these symptoms had subsided completely, she was discharged on day 30 post symptom onset while she was still LASV RNA positive in plasma. She was readmitted upon the reoccurrence of fever on day 35 when the first lumbar puncture was performed. The CSF RT-PCR tested positive on that day as well as on day 39. She first tested CSF negative on day 50, after which she was discharged again with low levels of LASV RNA detectable in plasma. Patient 11, a 7-year-old girl, presented to ISTH 9 days after symptom onset with fever, headache, vomiting, abdominal pain, and acute kidney and liver injury, as well as highly deranged inflammatory markers. The first CSF RT-PCR was performed on day 24 after the reported symptom onset with a positive result. She tested CSF negative on day 30 and plasma negative on day 36.

## DISCUSSION

In this retrospective laboratory-based study, we frequently observed LASV RNA in CSF of pediatric LF patients who underwent lumbar puncture due to CNS symptoms, primarily those of meningoencephalitis, as listed in Table 1. In some of these patients, the virus was not detected in plasma, and laboratory confirmation of LF required CSF testing. The viral load in CSF was often higher than in plasma, indicating a neuroinvasive infection with active virus replication in CNS.

Our observations raise the question of whether higher viral loads in CSF may be due to the CNS being an immune-privileged site with preferential viral replication or due to other causal mechanisms. Active immune evasion by downregulating the neural major histocompatibility complex class I expression and thereby reducing the effective immune response by cytotoxic T cells as in human immunodeficiency virus-1 (HIV-1) and cytomegalovirus infection should be investigated as potential mechanism in future studies [31–33]. Furthermore, it is conceivable that host factors, such as varying degrees of immunocompromise, may contribute to the likelihood of cerebral involvement [22].

The course of disease in patients 9 and 11, for whom the CSF RT-PCR was conducted several weeks after admission, might suggest that CNS manifestations occurred only in later stages of disease. This suggests that neuroinvasive LASV infection may also present with postacute sequelae. Virus latency in the CNS, dormancy in nerve ganglia, or autoimmune phenomena causing encephalitis present possible pathomechanisms in these patients. The 3 fatal cases had an opposite distribution of viral load compared to the other patients, with higher concentrations in plasma than in CSF. This proposes a different pathomechanism—likely an overwhelming systemic disease with loss of endothelial integrity including in the BBB.

Despite ongoing research, it remains unclear whether LASV is a neurotropic virus in humans. Viruses are known to target the brain in multiple ways, including inflammation (HIV, tick-borne encephalitis virus) [34, 35], direct neurotropic effects (herpes simplex virus [HSV], West Nile virus, Japanese encephalitis virus) [34–37], and thrombosis and bleeding (severe acute respiratory syndrome coronavirus 2 [SARS-CoV-2], varicella zoster virus [VZV]) [37, 38], as well as virus reactivation from dormancy state in the sensory nerve ganglia (HSV, VZV, Ebola virus) [19, 34, 37, 39]. For HSV type 1, which has been studied extensively, retrograde axonal transport along the trigeminal or olfactory nerve with spread to frontal and temporal lobes or hematogenous dissemination has been discussed [40]. Upon reactivation, HSV may infect other neurons by transmission of infectious viral particles [40]. Furthermore, an induction of autoimmune processes resulting from the immune response to the virus has been described. These lead to autoantibody production, particularly, but not limited to, antibodies against the *N*-methyl-D-aspartate receptor and to autoimmune encephalitis [41, 42]. For arenaviruses, different CNS entry pathways have been described, particularly a BBB crossing for LCMV [22, 23] and axonal transport for JUNV [24]. Dependent on host immunocompetence, viral load, and strain, both acute and chronic neurological sequelae may occur following LCMV and JUNV infection [22, 25]. In particular, virus-induced BBB permeability with trans- and paracellular CNS penetration, the “Trojan-horse method” [43], and (retrograde) axonal transport [24] require further investigation in LASV research as other members of the Arenaviridae family are suspected to utilize them [24, 43].

Our findings have implications for development of drugs against LF, and viral clearance from CSF should be considered in the design of respective clinical trials. Ribavirin, which is used as a standard yet off-label treatment for LF, has been shown to penetrate the BBB if administered intravenously in high concentrations in subacute sclerosing panencephalitis [44] and may therefore meet relevant pharmacokinetic criteria for use in CNS infections. Clinical trials are currently ongoing to evaluate other LF treatments including favipiravir [45], which showed low brain penetrance when tested against CNS manifestations of SARS-CoV-2 [46].

The main limitation of our study is the retrospective design based on existing data from routine clinical management. Although there are indications for lumbar puncture (Table 1), all findings are biased by the decisions made by the treating physicians. Thus, we could include only children in the analysis, because lumbar puncture was not conducted during the study period in adults. It is an invasive procedure that needs to be weighed carefully against potentially significant bleeding complications in patients with viral hemorrhagic fever. For these reasons, this study cannot provide estimates for the prevalence of LASV RNA detection in CSF in patients with LF. It might be ethically challenging to develop a prospective observational study protocol that involves lumbar puncture in all participants in an unbiased way, irrespective of a clinical indication.

Additionally, only general symptoms recorded at the time of patient sampling, based on information provided by the clinician completing the LF diagnostic request form, were available. Signs of meningitis and encephalitis, namely neck stiffness and focal deficits, were not among the symptoms routinely assessed on the RT-PCR request form. We tried to extract further clinical and laboratory information from patient files; however, the data were scattered and not suitable for scientific analysis. Leftover samples were not available for additional analyses. Furthermore, there is the seeming lack of hearing loss, a common neurological complication of LF, among our patients. This complication is more common in the subacute than the acute phase of illness, during which patients are usually hospitalized [6, 11, 47, 48]. In addition, hearing assessments in children are difficult because of the nonavailability of sensitive screening methods for children under the age of 5 and due to the challenges with follow-up assessments in older children. In conclusion, the design of this study did not allow to determine clinical manifestations or laboratory parameters associated with the presence of viral RNA in CSF.

Finally, we cannot present data on infectious particles in CSF. The available data were on LASV RNA only and the RT-PCR may have detected defective virus or free virus RNA.

To address the above-mentioned limitations, prospective studies in adults and children combining a structured clinical neurological examination and laboratory analysis of biomarkers in CSF and plasma are warranted. Multiplex PCR for the most common meningitis and encephalitis pathogens may rule out other infections as a rival cause for neurological symptoms. LASV immunoglobulin G and immunoglobulin M antibody testing will help to clarify in which phase of disease neurological symptoms arise. Viral sequencing may provide information on viral characteristics potentially associated with neuroinvasion. Postmortem minimally invasive tissue sampling will help to uncover how LASV gains access to the CNS and answer if and where the virus persists inside the CNS.

## CONCLUSIONS

Our results imply that in endemic areas, LF presents an important differential diagnosis for meningoencephalitis and has to be considered even in cases in which plasma PCR tests remain negative. To advance in clinical management and drug development, LF must be acknowledged as a neuroinvasive infection. The mechanisms by which LASV affects the CNS remain poorly understood, and further research is needed to determine why CNS manifestations occur in certain patients and at which stage of disease neuroinvasive infection arises.

## Notes

***Acknowledgments*.** We are grateful to the dedicated staff of the Lassa fever diagnostic laboratory at Irrua Specialist Teaching Hospital (ISTH), Nigeria, for their invaluable support in sample processing, diagnostics, and logistics. We also thank the clinical staff on the Lassa ward at ISTH for their unwavering commitment to patient care and for facilitating clinical data collection under challenging conditions. Their contributions were essential to the success of this study.

***Author contributions*.** H. C. S. M. worked on the design of the study; acquisition, analysis, and interpretation of data; and drafting and revision of the submitted article. C. O. E. was involved in the conception and design of the study, acquisition of data, and drafting and revision of the submitted article. J. O., Ri. E., E. B. O., M. H., J. M., I. F. O., M. A., E. E., I. H. O., C. O. U., J. O. I.-E., K. O. I., and O. I. E. helped with the acquisition, analysis, and interpretation of data. U. M. A., C. H., D. A., Th. O., I. O., S. O., E. O.-E., Re. E., and C. N. E. took part in the acquisition of data. M. P., S. O. I., A. E. A., L. O. A., and D. A. worked on the acquisition of data and the revision of the article. S. G., M. R., L. O., Ti. O., and G. A. were involved in the conception and design of the study; acquisition, analysis, and interpretation of data; and the drafting and revision of the article.

***Prior presentations.*** Parts of this work were presented at the Conference on Tropical Medicine and Global Health, held 19–21 September 2024, in Düsseldorf, Germany.

***Data availability*.** The code used to analyze the Wilcoxon signed-rank test is available at https://github.com/hannahmuellerbnitm/LASV_CSF_retrospective/ (accessed 7 July 2025).

***Financial support.*** This work was supported by the German Research Foundation (grant numbers GU 883/4-2 to S. G. and Collaborative Research Center 1648/1 grant number 2024–512741711 “Emerging Infections” to M. R., Ti. O., and S. G.; iD fellows programme grant number 493624519 to Ti. O.); the Leibniz Association (grant numbers J103/2020 to T. O. and J59/22018 to L. O.); and the Global Health Protection Programme of the German Federal Ministry of Health (grant numbers FKZ 2523GHP007 to M. R. and ZMI5 2523GHP006 to S. G.).
